# Acute Appendicitis in a Child With Acute Leukemia and Chemotherapy-Induced Neutropenia: A Case Report and Literature Review

**DOI:** 10.7759/cureus.8858

**Published:** 2020-06-27

**Authors:** Alaa Ali, Saeed Alhindi, Adel A Alalwan

**Affiliations:** 1 Pediatric Surgery, Salmaniya Medical Complex, Manama, BHR; 2 Nephrology, Salmaniya Medical Complex, Manama, BHR

**Keywords:** appendicitis, chemotherapy-induced neutropenia, acute leukemia

## Abstract

Acute appendicitis is a rare but important complication in children with leukemia. It can be difficult to diagnose, and it has a complicated disease course, especially in patients receiving chemotherapy. Awareness of these complications is critical, particularly in cases where surgical intervention is required. We report a child with T-cell acute lymphoblastic leukemia and chemotherapy-induced neutropenia who presented with acute appendicitis. He was successfully treated with broad-spectrum empiric antibiotics and a delayed laparoscopic appendectomy.

## Introduction

Children with leukemia, especially those treated with chemotherapy, are vulnerable to a variety of infections that result from bone marrow suppression and neutropenia. Intra-abdominal and right lower quadrant (RLQ) infectious complications, in particular, are often sources of severe sepsis and result in an increased mortality rate [1]. Acute appendicitis is a primary cause in these cases, but its presentation is often aberrant, increasing the challenges in diagnosis and management. The optimal treatment approach for these cases of acute appendicitis remains an area of ongoing controversy [2,3]. However, studies describing acute appendicitis in children with hematological malignancies are scarce, and available information originates largely from a limited number of retrospective studies and case reports [2,4,5].

## Case presentation

A 10-year-old boy with T-cell acute lymphoblastic leukemia (T-ALL) and chemotherapy-induced neutropenia presented with a two-day history of abdominal pain, vomiting, and fever. The pain, which commenced gradually, was intermittent and dull, and the patient sought relief by lying still. Though initially localized to the periumbilical area, the pain migrated to the RLQ the day after onset, and it was associated with several episodes of vomiting containing no bile or blood. The patient reported feeling feverish with chills and a decreased appetite. He denied a history of sore throat, cough, shortness of breath, diarrhea, or dysuria.

The patient was diagnosed two months earlier with T-ALL after presenting with an anterior mediastinal mass. He was treated with chemotherapy according to the Berlin‐Frankfurt‐Munster protocols [6]. The consolidation phase of treatment was started after completing a four-week course of induction therapy. The most recently scheduled chemotherapy (cyclophosphamide, cytarabine, mercaptopurine, intrathecal methotrexate) was received five days before presenting to our hospital.

After admission, the patient had a fever, with a maximum recorded temperature of 38.5°C. An examination revealed that the patient was mildly dehydrated and had a blood pressure of 100/70 mmHg and a heart rate of 85 beats/min. Deep palpation during a serial abdominal examination revealed tenderness at the right iliac fossa but no peritoneal signs. The abdomen was not distended, and guarding, rigidity, and rebound tenderness were not evident. Bowel sounds were present. The remainder of the examination was unremarkable.

A complete blood count at admission revealed pancytopenia (Table 1), and severe neutropenia (68 cells/μL). The C-reactive protein was elevated (83.9 mg/L). The coagulation profile, urea, creatinine, electrolytes, and liver function tests were within normal ranges. Urinalysis was negative as were blood and urine cultures.

**Table 1:** Complete blood count at admission and on the sixth day of hospitalization. WBC, white blood cell; L, low; MCV, mean corpuscular volume; ANC, absolute neutrophil count; H, high.

Parameter (Reference Range)	Results at Admission	Results on Day 6
WBC (3.6-9.6 x 10^9^/L)	1.13 x 10^9^/L (L)	8.16 x 10^9^/L
Hemoglobin (12-14.5 g/dL)	9.7 g/dL (L)	11.2 g/dL (L)
Hematocrit (33%-45%)	29.3% (L)	31.7% (L)
MCV (80-97 fL)	85.2 fL	84.2 fL
Platelets (150-400 x 10^9^/L)	32 x 10^9^/L (L)	113 x 10^9^/L (L)
Neutrophils (40%-70%)	6% (L)	63%
ANC (≥1,500 cells/μL)	68 cells/μL (L)	5,141 cells/μL
Lymphocytes (20%-50%)	92% (H)	33.5%
Blast cells (<1 %)	Nil	Nil
Monocytes (1.5%-9%)	2%	2.8%
Eosinophils (<4%)	Nil	0.1%
Basophils (<2%)	Nil	0.4%

**Figure 1:**
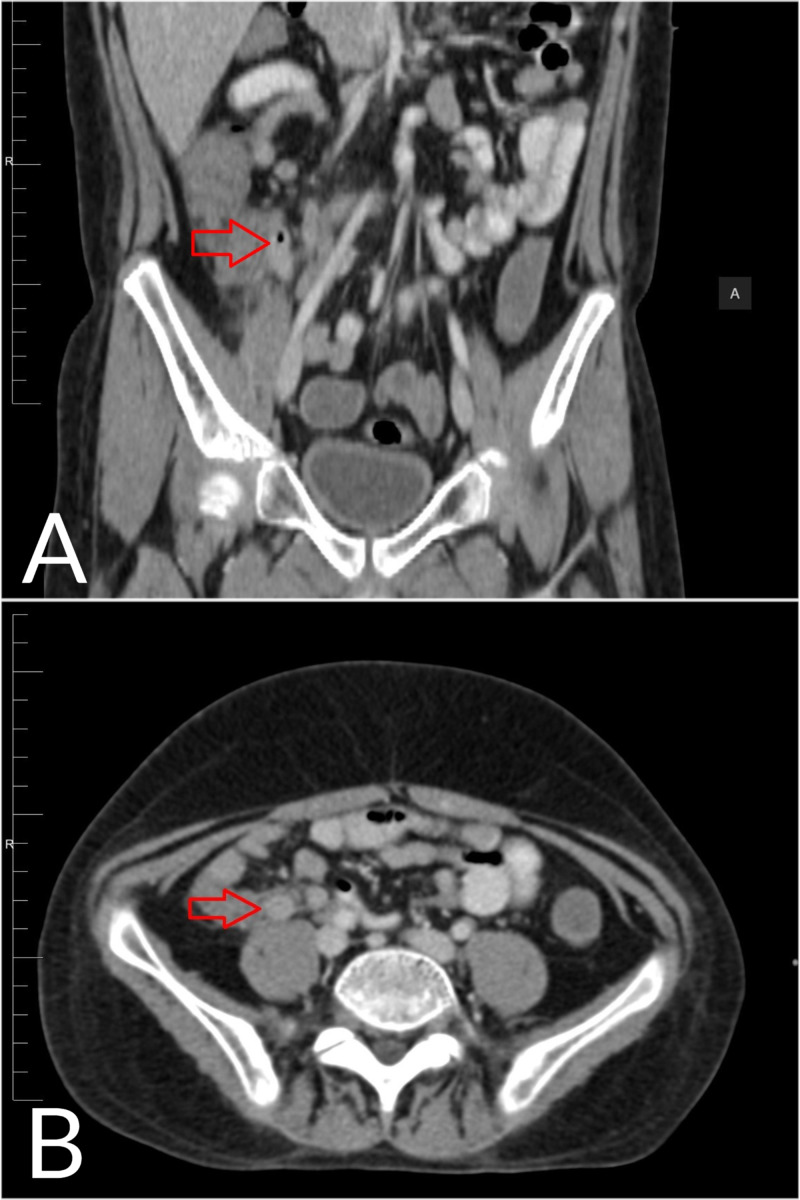
CT of the abdomen. (A) Coronal CT scan image. (B) Axial CT scan image. Images show a dilated, thick-walled appendix measuring up to 7 mm (red arrows) with peri-appendicular inflammatory fat stranding and minimal free fluid.

Ultrasound revealed a blind-ended non-compressible dilated appendix measuring 7 mm in diameter accompanied by peri-appendicular edema and probe tenderness. These findings were confirmed by CT (Figure 1), establishing a diagnosis of acute appendicitis.

The patient was managed conservatively with intravenous antibiotics, proper hydration, and analgesics. Employing empirical coverage for febrile neutropenia, a seven-day course of antibiotics was started that included piperacillin-tazobactam (3,800 mg three times daily), gentamicin (80 mg three times daily), and metronidazole (330 mg four times daily). Appendectomy was deferred until the bone marrow recovered from chemical suppression. Both filgrastim (300 mcg daily by subcutaneous injection) and platelet transfusion were required to correct pancytopenia. After six days of hospitalization, neutrophil count normalized with an increase in hemoglobin and platelet levels (Table 1).

**Figure 2:**
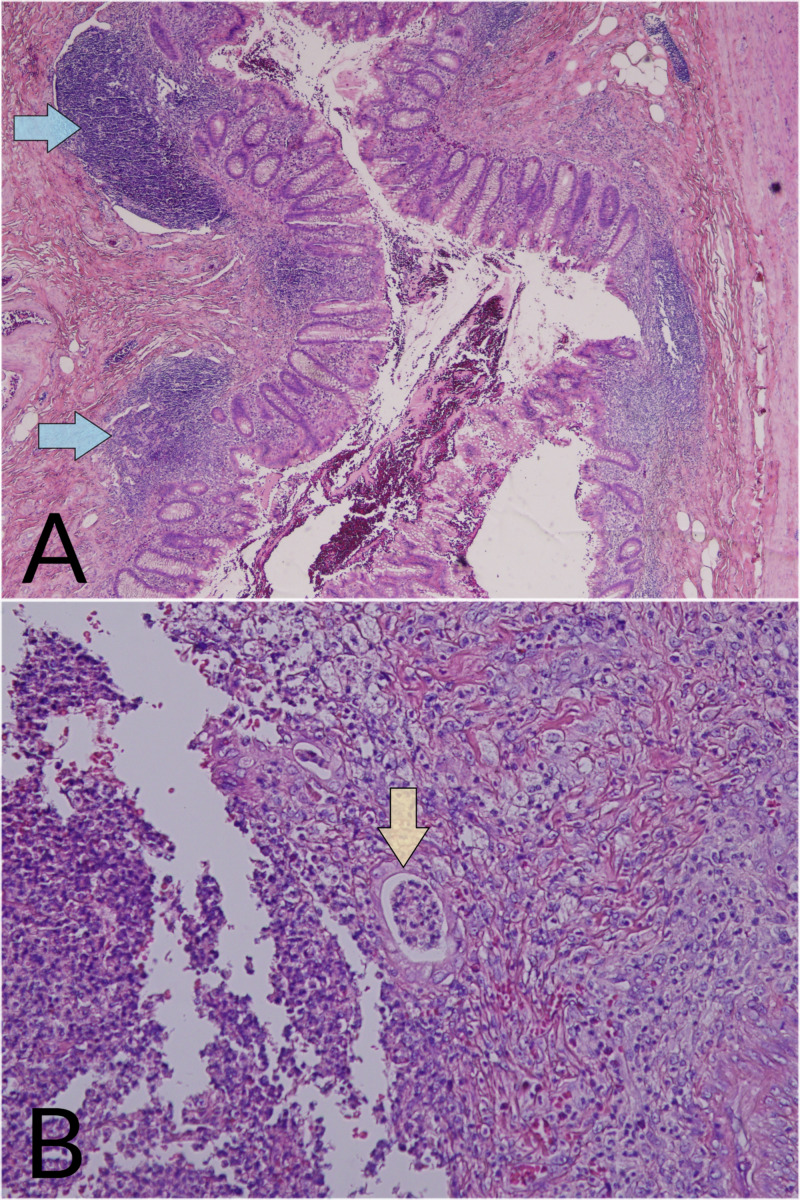
Hemotoxylin and eosin (H&E)-stained section of the appendix. (A) Low power view (H&E, original magnification x40). The image shows an appendiceal mucosa with mucosal erosions, submucosal lymphoid aggregates (blue arrows), and transmural acute inflammatory cell infiltrate. (B) Higher power view (H&E, original magnification x100). The mucosa shows ulcerations with cryptitis and crypt abscesses (yellow arrow).

Although the patient did not deteriorate during hospitalization, RLQ abdominal pain with tenderness on physical examination recurred. He underwent a laparoscopic appendectomy the day after neutropenia resolved. An acutely inflamed appendix was removed with no immediate intraoperative complications. The tip of the appendix was adherent to the cecum and lateral abdominal wall. The appendix was not gangrenous nor perforated, and no purulent material was noted. The histopathological examination of the appendix revealed focal mucosal ulcerations and transmural neutrophilic infiltration, but no leukemic cells were observed (Figure 2).

The patient was vitally and clinically stable after surgery. On the second postoperative day, he was ambulatory and tolerating food. He did not report fever, vomiting, or abdominal pain. He was discharged on the fourth postoperative day with close follow-up at the pediatric surgery and hematology outpatient clinics.

## Discussion

Appendicitis is the most common reason for surgery in the pediatric population, affecting 1% of all children below 15 years of age [2]. However, those with hematological malignancies have a relatively low incidence of appendicitis. Studies generally report a rate of 0.5% to 1.5% [4,5,7]. The etiology of appendicitis in patients with leukemia is similar to that in the general population: most cases are secondary to a luminal obstruction by a fecalith or lymphoid hyperplasia. Nevertheless, rare cases have been attributed to direct leukemic cell infiltration to the appendix [8,9].

With hematological malignancies, acute appendicitis can present with atypical signs and vague discrete symptoms such as non-localized abdominal pain, abdominal distention, fever, and diarrhea. A physical examination can include diffuse and RLQ abdominal tenderness without peritoneal signs [4,5,7]. Based on clinical findings alone, acute appendicitis can be difficult to distinguish from other surgical conditions encountered in children with leukemia such as typhlitis, ileocecal intussusception, intestinal obstruction, pancreatitis, and others [4]. In addition, chemotherapeutic agents complicate the diagnosis of acute appendicitis by masking the signs and symptoms, and their toxic effects can mimic the acute surgical abdomen [10,11]. In many cases, the diagnosis can be delayed or missed, thus increasing the morbidity and mortality rates.

Many times, a diagnosis of acute appendicitis requires imaging studies in neutropenic patients. Abdominal ultrasounds and CT scans are reportedly appropriate in these patients [4,5,12]. A CT scan is especially important so that appendicitis can be differentiated from neutropenic colitis (typhlitis) because their clinical presentations can be nearly indistinguishable. Using CT, the findings of acute appendicitis include a dilated appendix, appendicular wall thickening, and peri-appendicular fluid. In contrast, typhlitis is indicated by right-sided colon wall thickening, pericolonic stranding, ascites, and cecal pneumatosis [10]. On rare occasions, typhlitis mimics appendicitis on imaging when it is limited to the appendix and lacks cecal involvement. In these cases, a definitive diagnosis requires biopsy [11]. Appendicitis is histologically characterized by an intense inflammatory reaction involving some or all layers of the appendix accompanied by a significant neutrophil infiltrate [13]. On the other hand, typhlitis is associated with submucosal edema and diffuse necrosis without a large number of neutrophils [14]. Our patient had the typical CT findings of appendicitis, and his biopsy showed a predominantly neutrophilic infiltrate despite presenting with severe neutropenia.

The management of acute appendicitis in children with leukemia and chemotherapy-induced neutropenia is debatable, and the timing of surgical intervention remains controversial [10]. Some authors suggest non-operative conservative management, but others advocate early appendectomy as the treatment of choice. The conservative approach includes general supportive measures and administration of adequate broad-spectrum antibiotics. This approach was recommended by Wiegering et al., who observed that even perforated appendicitis could improve with conservative care alone [12]. Conservative management avoids the risks associated with postoperative complications in these patients. However, a longer period is necessary for complete recovery, and the chances for clinical worsening, perforation, and recurrence are greater [15]. A delay in recovery from appendicitis can also lead to a delay in resuming chemotherapy, which can worsen the patient’s underlying illness and escalate medical costs [2].

Several authors noted lower rates of complications and mortality with early surgical treatment of acute appendicitis in children with leukemia. Angel et al. reported a series of 14 pediatric patients with leukemia, 13 of whom received appendectomies, and one received conservative treatment. The latter died. Of the surgical patients, nine had uneventful recoveries, three had complications (including infection and intraoperative bleeding), and one died [4]. In several recent studies, neither operative morbidity nor mortality was observed following open or laparoscopic appendectomies [2,3,5]. Moreover, these studies support early surgical intervention in leukemic patients with appendicitis because symptoms are resolved more quickly and hospital stays are shorter [3]. Laparoscopic appendectomy appears to be the preferred procedure, resulting in a small wound, fewer wound infections, less pain, rapid recovery, and lower risk of hemorrhagic complications [4,16].

Higher rates of infectious perioperative complications are expected in neutropenic patients, and postoperative mortality rates have been reported as high as 41% [17]. Thus, a combined treatment approach was used in our patient, implementing empiric antibiotic therapy initially and delaying surgical intervention until the absolute neutrophil count reached a cutoff of 1,500 cells/μL. The patient was monitored daily for signs of deterioration that might require emergency appendectomy. Scarpa et al. retrospectively compared the combined treatment approach with conservative medical management and early surgical intervention in neutropenic children with appendicitis. Of 30 children treated for cancer (mean age, 8.8 years), 90% had hematological malignancies. The combined approach was used in 17 cases, and delayed appendectomy was successful. Conservative treatment was used in seven cases and early surgical intervention in six cases. All the therapeutic approaches were successful, and transitory complications were noted in only three patients. The length of hospital stay (mean, 20 days) was not associated with the treatment method [15]. 

## Conclusions

Acute appendicitis in children with leukemia and chemotherapy-induced neutropenia often presents atypically. Correct and early diagnosis is essential and has a direct impact on treatment. Abdominal ultrasounds and CT scans provide valuable diagnostic support; nevertheless, a histopathological assessment of the appendix can be required.

Our observations support the effectiveness of a combined approach in the treatment of appendicitis in these cases, employing broad-spectrum antibiotic therapy and a delayed appendectomy. However, continuous monitoring for signs of deterioration that might indicate the need for an emergency surgical intervention is required. In these patients, the laparoscopic surgical approach seems to limit the risk of postoperative complications and shortens the hospital stay.
